# Effects of hirudotherapy on liver functions, lipid profile, and insulin sensitivity in rats with metabolic syndrome

**DOI:** 10.1186/s12906-025-05241-x

**Published:** 2026-01-08

**Authors:** Alican Bilden, Seda Koçak, Halime Tozak Yıldız, Fatih Çakır, Merve Kahraman, Muttalip Çiçek

**Affiliations:** 1 Faculty of Medicine, Department of Medical Parasitology, Kırşehir Ahi Evran University, Kırşehir, Türkiye; 2 Faculty of Medicine, Department of Physiology, Kırşehir Ahi Evran University, Kırşehir, Türkiye; 3 Faculty of Medicine, Department of Histology and Embryology, Kırşehir Ahi Evran University, Kırşehir, Türkiye; 4 Faculty of Dentistry, Department of Microbiology, Batman University, Batman, Türkiye

**Keywords:** Metabolic syndrome, Hirudotherapy, Therapeutic effect, Alternative treatment

## Abstract

**Background:**

Metabolic syndrome (METS) is a multifactorial cardiometabolic disorder characterized by abdominal obesity, insulin resistance, hypertension, dyslipidemia, and hyperglycemia, substantially increasing the risk of type 2 diabetes and cardiovascular diseases. The variable effectiveness of lifestyle and pharmacological interventions has heightened interest in complementary approaches. Hirudotherapy, containing a wide range of bioactive compounds, may offer therapeutic benefits. This study aimed to evaluate the effects of hirudotherapy on metabolic parameters in a rat model of METS.

**Methods:**

Twenty-four male Wistar rats were divided into four groups: Control (*n* = 6), METS (*n* = 6), METS + 4-week hirudotherapy (*n* = 6), and METS + 8-week hirudotherapy (*n* = 6). METS was induced through a modified high-fat and fructose-enriched diet. The effects of hirudotherapy on liver enzymes (AST, ALT), lipid profile (TG, LDL cholesterol, HDL cholesterol), hemodynamic parameters (systolic/diastolic blood pressure, mean arterial pressure, heart rate), glucose metabolism (OGTT, AUC), and liver histopathology were assessed. Pellet and water intake were monitored to evaluate possible influences on appetite regulation.

**Results:**

Hirudotherapy demonstrated hepatoprotective activity, yielding significant reductions in AST and ALT levels (*p* < 0.05). TG levels increased in METS, while LDL cholesterol showed partial improvement and HDL cholesterol remained unchanged. Systolic blood pressure significantly decreased in the hirudotherapy-treated groups, with no significant differences in diastolic pressure or heart rate. OGTT and AUC analyses revealed improved glucose tolerance and reduced hyperglycemia following hirudotherapy (*p* < 0.05). Histopathology showed marked improvements in steatosis, sinusoidal dilatation, and hydropic degeneration, although minor hemorrhagic foci persisted. Differences in pellet consumption suggested a potential regulatory effect on appetite and metabolic balance.

**Conclusion:**

Hirudotherapy may exert beneficial effects in METS by improving liver function, modulating lipid metabolism, enhancing insulin sensitivity, and reducing inflammation. Its influence on food intake may further support metabolic homeostasis. These findings support hirudotherapy as a potential biotherapeutic approach and warrant further mechanistic and clinical investigations.

## Introduction

 Metabolic syndrome (METS) is a significant modifiable risk factor for cardiovascular disease, type 2 diabetes, and other health complications, with its global prevalence rapidly increasing due to urbanization, sedentary lifestyles, and changes in dietary habits [1]. The etiology of METS is associated with complex mechanisms such as genetic predisposition, insulin resistance, dysfunctional adipose tissue accumulation, abdominal obesity, ectopic lipid deposition, systemic inflammation, and dyslipidemia. These mechanisms manifest as clinical symptoms, including abdominal obesity, hyperglycemia, dyslipidemia, and hypertension [2]. The increasing prevalence of METS, alongside obesity epidemics, affects approximately one-quarter of the global population, increasing the risk of chronic diseases and imposing a significant burden on healthcare systems [3].

Current METS management strategies focus on lifestyle modifications such as diet, physical activity, and visceral fat control. However, due to individual metabolic response variations, genetic factors, and the complex nature of the disease, these approaches do not always yield the desired outcomes for every individual and are not entirely effective in preventing METS-related diseases. Therefore, the challenges in METS treatment highlight the potential supportive role of alternative and innovative medical approaches [1, 2, 4].

Traditional and complementary medicine practices have been used throughout history in various cultures worldwide for the treatment of different diseases [5]. In this context, hirudotherapy (leech therapy) emerges as a potential complementary treatment for managing circulatory disorders, microvascular dysfunction, and inflammatory processes associated with METS. The bioactive compounds present in leech saliva, including hirudin, eglins, bdellins, and other active molecules, exhibit anticoagulant, anti-inflammatory, and microcirculation-enhancing effects. These properties may contribute to alleviating vascular dysfunction, chronic inflammation, and circulatory impairments caused by METS [6, 7].

Hirudotherapy has been used throughout history in both human disease treatment and veterinary medicine and has recently regained significance as a supportive method in modern medicine [5]. Today, hirudotherapy is widely used by plastic surgeons, particularly in reconstructive surgery, to prevent venous congestion and support tissue regeneration. Factors such as antibiotic resistance, the demand for minimally invasive procedures, and the advancement of personalized medicine have further increased interest in hirudotherapy [8]. Therefore, the potential effects of hirudotherapy and other traditional medical approaches in the treatment of METS should be further investigated.

This study aims to scientifically evaluate the therapeutic effects of hirudotherapy on METS using an experimental animal model. Specifically, it investigates the potential role of bioactive compounds in leech saliva in managing vascular dysfunction and inflammation associated with METS, with the goal of providing new insights into the efficacy of alternative medical approaches in METS treatment.

## Methodology

### Animal selection and experimental conditions

This study was conducted at the Experimental Animals Research Center of Kırşehir Ahi Evran University, where a total of 24 adult male Wistar albino rats (220 ± 20 g) were used. Prior to and throughout the experiment, all animals were housed in a controlled environment maintained at 22–24 °C with a 12-hour light/12-hour dark cycle. The rats were provided standard pellet feed and tap water *ad libitum*, with no restrictions on food or water intake. During the first 10 weeks of the experiment, the animals were housed in groups of three per cage; after the induction of the metabolic syndrome model and the initiation of hirudotherapy sessions, each rat was transferred to an individual cage.

To minimize acute stress related to restrainer exposure during experimental procedures, all rats underwent a brief acclimatization period lasting 3–5 min before each session. To ensure animal welfare and procedural consistency, all rats—including those in the control and METS groups—were placed in the same type of standard acrylic restrainer device (internal dimensions: 25 cm length × 7 cm diameter) during the application period. This standardization ensured that any potential stress effects associated with restrainer use were experienced equally across all groups and helped prevent restrainer-related experimental bias.

All procedures were carried out in accordance with the ARRIVE guidelines, and the study protocol was approved by the Local Ethics Committee for Animal Experiments of Kırşehir Ahi Evran University (Approval No: 07/12/2023-23−2, Türkiye). All measures necessary to ensure animal welfare were strictly implemented throughout the study.

### Euthanasia procedure

At the conclusion of the experimental period, euthanasia was performed via intraperitoneal administration of sodium thiopental at a dose of 50 mg/kg. To confirm death, thoracotomy was subsequently conducted. Humane endpoints were established to minimize animal suffering and included the onset of severe respiratory distress, irreversible loss of mobility, or overt signs of pain or discomfort. In such cases, animals were promptly euthanized without delay. Confirmation of death was based on the absence of cardiac activity, respiratory movements, and pupillary reflex.

### Procurement, maintenance conditions, and application protocol of medicinal leeches

The medicinal leeches used in this study belonged to the species *Hirudo verbana* and were obtained from the Medicinal Leech Breeding Laboratory of Kırşehir Ahi Evran University, Faculty of Medicine. The leeches used for the applications weighed between 1.5 and 2 g on average. Prior to use, they were starved for 3–6 months to ensure physiological adaptation. Throughout the study, all leeches were kept in well-aerated plastic containers filled with dechlorinated tap water; the containers were maintained at a constant room temperature of 22–24 °C, and the water was changed every two days according to a standardized protocol.

**Fig. 1:**
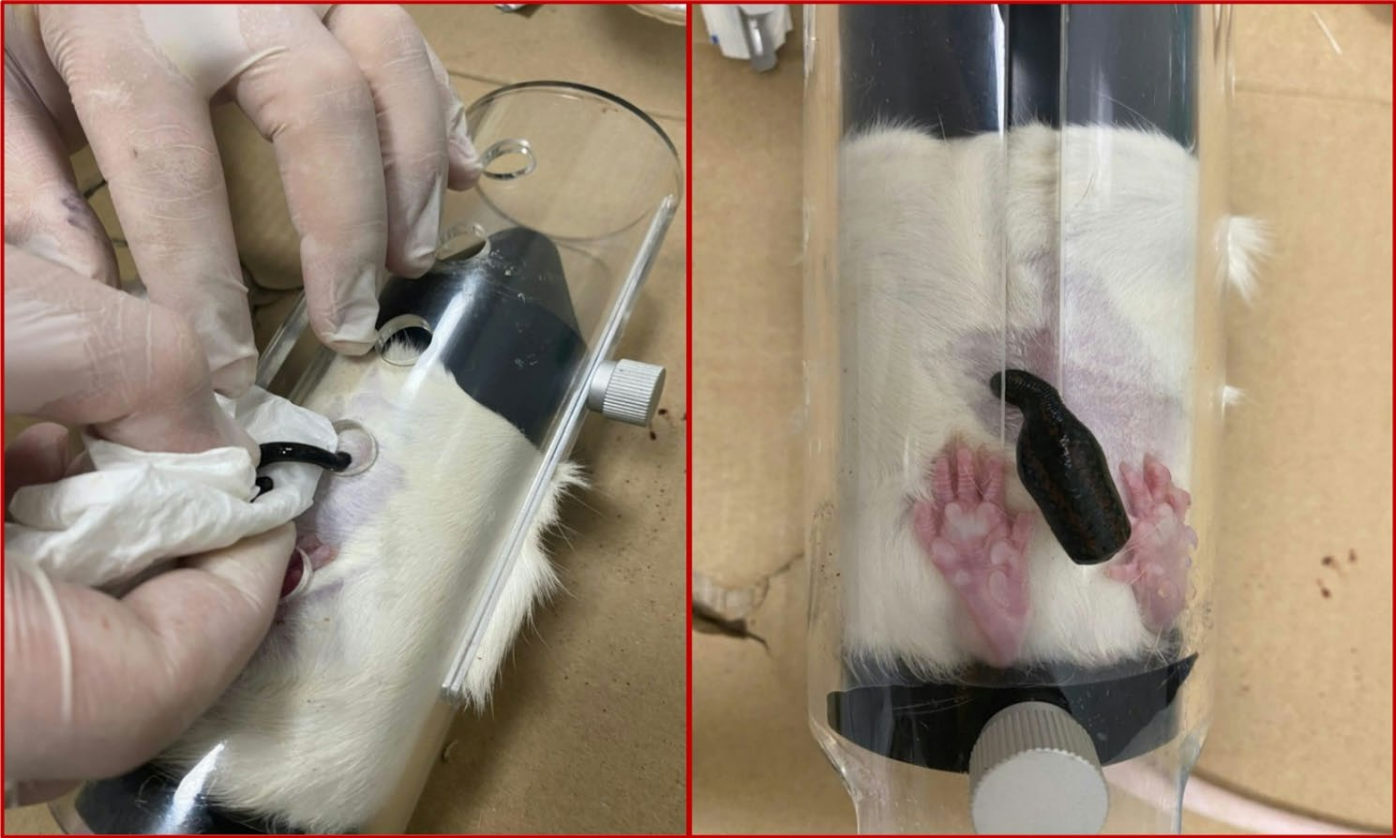
Application of hirudotherapy in rats

During the hirudotherapy procedure, a single leech was placed on the shaved peritoneal region of each rat, and restrainer cages were used to limit animal movement during the session (Fig. 1). The criterion for a successful application was defined as the leech attaching spontaneously to the treatment site and feeding continuously for 15 min. Because the natural feeding duration of leeches can vary between 10 and 25 min, a fixed 15-minute application period was selected to reduce biological variability and ensure temporal standardization across groups. This standardization was adopted to enhance reproducibility under controlled experimental conditions.

If a leech failed to attach, detached before completing the required duration, or did not sustain feeding behavior, the attempt was considered unsuccessful, and the procedure was restarted using a new leech.

In accordance with national and international regulations governing clinical hirudotherapy, all leeches used during the applications were considered single-use biological material after treatment. They were disposed of following appropriate medical waste management procedures, in compliance with the Traditional and Complementary Medicine Practices Regulation [9]. This approach aligns with the FDA’s 2004 approval regarding the use of leeches in microsurgery and plastic surgery, emphasizing the prevention of reuse and ensuring biosafety [10].

### Experimental groups

A total of 24 male Wistar albino rats were divided into four experimental groups:

#### Group 1 - Control group (*n* = 6)

Rats in this group were provided standard rat chow (20% kcal protein, 10% kcal fat) and tap water throughout the experimental period.

#### Group 2 - METS group (*n* = 6)

To induce the metabolic syndrome model, rats were fed a modified high-carbohydrate + high-fat diet (HCD + HFD) containing high fructose and high fat. 60% of total energy intake was derived from fat, and the elevated carbohydrate (fructose-rich) content ensured full alignment with the HCD + HFD model. This specialized diet was administered *ad libitum* for 18 weeks.

#### Group 3 - METS + 4TH WEEK hirudotherapy (*n* = 6)

Rats in this group received the same high-carbohydrate + high-fat (HCD + HFD) diet as the MetS group. The dietary regimen lasted for a total of 14 weeks. Hirudotherapy was initiated after completion of week 10 of the modified diet and was applied once per week for 4 consecutive weeks. Treatment sessions were conducted during weeks 11, 12, 13, and 14, with each session lasting 15 min.

#### Group 4 - METS + 8TH WEEK hirudotherapy (*n* = 6)

Rats in this group were also maintained on the HCD + HFD regimen. The diet was continued for a total of 18 weeks. Hirudotherapy was initiated after completion of week 10 and was administered once per week for 8 consecutive weeks. Treatment sessions were carried out during weeks 11 through 18, and each session lasted 15 min.

At the end of the experimental period, in vivo assessments for each group were completed 24 h after the final intervention. To achieve deep anesthesia, thiopental was administered at a dose of 50 mg/kg, followed by thoracotomy, during which the aorta was severed and blood samples were collected from the thoracic cavity. Liver samples designated for histological analysis were preserved in appropriate fixation solutions, while serum samples were stored at −80 °C for subsequent biochemical analyses. No unnecessary euthanasia was performed on the experimental animals throughout the study. The data obtained from each group were compared between control and treatment groups to evaluate the potential effects of metabolic syndrome and hirudotherapy.

### Feeding regimen of the animals

The induction of metabolic syndrome (METS) commonly relies on high-fat diets (HFD), which have been shown in various studies to trigger metabolic disturbances such as hyperglycemia, insulin resistance, dyslipidemia, and elevated circulating free fatty acids. Moreover, the literature reports that high-carbohydrate and high-fat dietary combinations (HCD + HFD) more accurately mimic the human METS phenotype compared with high-fat diet al.one [11, 12]. Therefore, in the present study, a modified high-fructose + high-fat diet was used to establish the MetS model.

The dietary formulation was adapted from the widely referenced D12451 high-fat diet protocol [13]. The components of the modified diet included casein, DL-methionine, L-cysteine, corn starch, maltodextrin, cellulose, butter, corn oil, fructose, mineral mix (10026), dicalcium phosphate (DCP), calcium carbonate, potassium citrate, vitamin mix (V10002), and choline. Owing to its elevated fat and carbohydrate (high-fructose) content, this formulation met the criteria for an HCD + HFD model.

The fat content of high-fat diets is reported in the literature to range between 30% and 70% [14]. Accordingly, to ensure the effective and sustainable induction of the METS model, the dietary fat percentage in this study was set at 60%. Throughout the experimental period, all animals had unrestricted access to water; the METS group received the experimental diet ad libitum, whereas the control group was provided standard pellet chow.

### Pellet and water consumption

Pellet consumption was monitored beginning after the first 10 weeks, once the METS model had been established and hirudotherapy interventions were initiated. Throughout the experimental period, pellet and water intake were measured twice per week, and the mean values were compared across groups. For each rat, food and fluid consumption were calculated by subtracting the remaining amount in the cage from the total amount provided. The resulting data were used to evaluate differences in feeding behavior and fluid intake among the experimental groups.

### Serum biochemical analyses

Collected blood samples were left to stand in tubes for 30 min and then centrifuged at 3,000 rpm for 10 min. Non-hemolyzed serum samples were promptly transferred to Eppendorf tubes and stored at −80 °C for biochemical analyses. To assess liver function, lipid profile, and metabolic parameters in rat serum, biochemical analyses were conducted. AST (Otto Scientific, OttoBC127), ALT (Otto Scientific, OttoBC128), TG (Otto Scientific, OttoBC155), HDL (Otto Scientific, OttoBC144), and LDL (Otto Scientific, OttoBC145) levels were measured using a MINDRAY-BS400 autoanalyzer with a colorimetric method based on the ELISA technique.

### Blood pressure measurement

To evaluate hemodynamic parameters among the groups, blood pressure measurements were performed using a tail cuff plethysmography system (MAY NIBP250, Turkey) following the final leech application. During the measurements, all animals were placed in a restraint tube for 20 min after completing the acclimatization process, and a cuff was attached to their tails.

For each animal, a total of five measurements were taken at one-minute intervals. The highest and lowest values were excluded, and the average of the remaining three measurements was used to calculate the following parameters:

- Systolic Blood Pressure.
- Diastolic Blood Pressure.
- Heart Rate.
- Mean Arterial Pressure (MAP).

Mean arterial pressure was calculated using the following formula:

MAP = Diastolic Pressure + (Systolic Pressure - Diastolic Pressure)/3.

The data obtained using this method were used to evaluate blood pressure variations among the experimental groups.

### Oral glucose tolerance test (OGTT)

The oral glucose tolerance test was performed at the end of experiments on rats that had been fasted for 12 h. Before glucose administration (0-minute), blood glucose levels were measured using an ACCU-Check^®^ device with blood samples collected from the tail veins. Subsequently, rats were administered 2 g/kg glucose solution via oral gavage. Following glucose loading, blood glucose levels were measured and recorded at 30, 60, 90, and 120 min. The collected data were analyzed to assess glucose tolerance levels in the rats.

### Histopathological analysis and scoring

Liver tissue samples obtained from all subjects were fixed in 10% formaldehyde solution for at least 72 h for light microscopic examination. Following fixation, the tissue samples were placed in cassettes and washed under running water for 24 h. To remove water, the tissues were passed through a graded series of alcohol solutions (50%, 70%, 80%, 90%, and 100%). For the clearing process, the tissues were treated with xylene and then embedded in melted paraffin. Sections 5–6 μm thick were prepared from the paraffin blocks and stained using the Hematoxylin-Eosin (H&E) staining method (Bio-Optica 05–06004/L Harris’ Hematoxylin & Bio-Optica 05–10002/L, Eosin Y 1%). Histological changes in the liver tissue architecture were microscopically evaluated using H&E staining. Each damage parameter was assessed in 20 different fields per liver section, and the mean percentage values were calculated for each group. Histopathological changes were graded as follows: 1 (mild) if observed in less than 33% of the liver cells and periacinar region, 2 (moderate) if observed in 33–66%, and 3 (severe) if observed in more than 66% of the tissue (none = 0, mild = 1, moderate = 2, severe = 3).

### Statistical analysis

All statistical analyses were performed using GraphPad Prism software (GraphPad Prism for Windows v5, 2007). The Shapiro-Wilk test was applied to assess the distribution of the data. Based on the results of the normality test, parametric tests were chosen for group comparisons, considering the quantitative nature of the data and assumptions for parametric tests. One-way analysis of variance (One-Way ANOVA) was used to determine differences among the four groups. Two-way ANOVA test was used in the analysis of repeated measurements. If a significant difference was found in the ANOVA, Tukey’s post-hoc test was applied to identify the source of variation. Results are presented as Mean ± Standard Deviation (Mean ± SD). A significance level of *p* < 0.05 was considered statistically significant.

## Results

### Evaluation of rat body weights

**Fig. 2:**
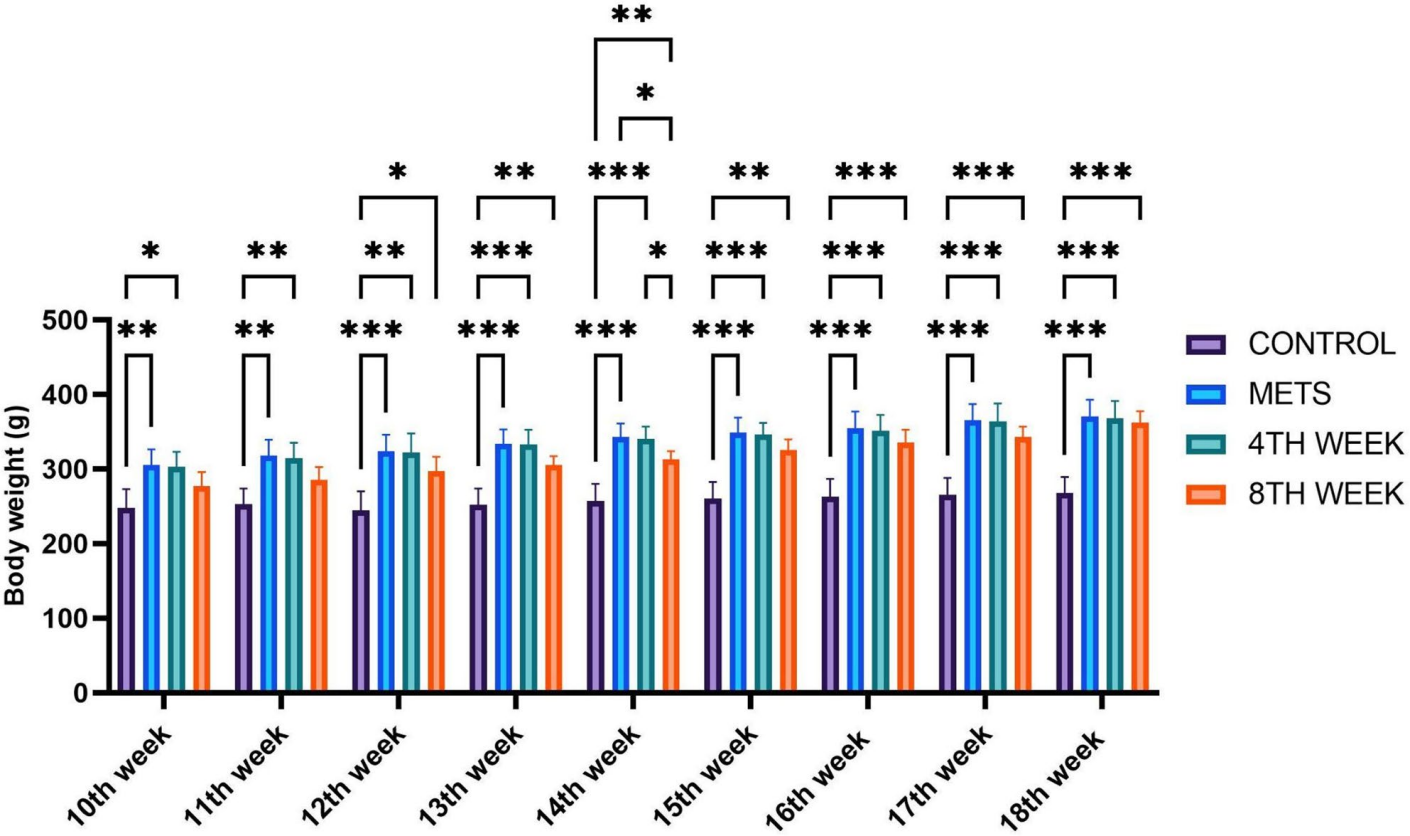
Weekly assessment of animal body weights. Data are represented as mean + SD. * < 0.05 ** < 0.01 ***< 0.001

Following the induction of METS, rat body weights were monitored for 10 weeks. At the 10th-week measurement, the average body weight of the METS group (305.3 g) was significantly higher compared to the control group (247.8 g). However, no statistically significant difference was observed between the METS group and the 4TH WEEK or 8TH WEEK hirudotherapy groups at this time point. At the 14th week, the 4TH WEEK (340.0 g) and 8TH WEEK (313.3 g) hirudotherapy groups, along with the METS group (342.7 g), had significantly higher body weights compared to the control group (257.3 g). By the 18th week, no statistically significant difference was detected between the 4TH WEEK (367.7 g) and 8TH WEEK (361.8 g) hirudotherapy groups, while their body weights remained comparable to the METS group (370.2 g) but significantly higher than the control group (267.8 g) (Fig. 2).

### Evaluation of pellet and water consumption

**Fig. 3:**
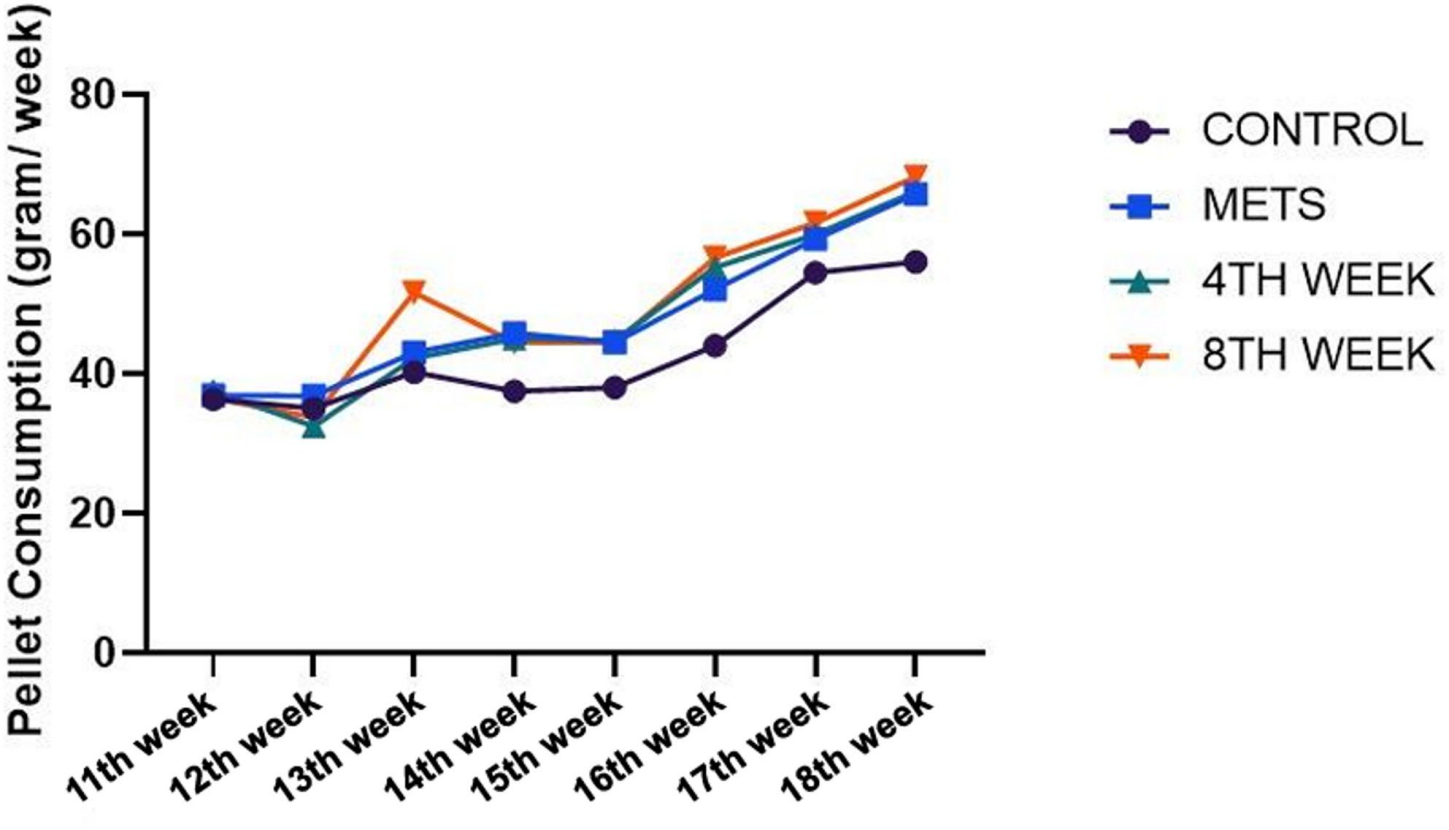
Evaluation of pellet consumption among groups. Data are represented as mean + SD. * < 0.05 ** < 0.01 *** < 0.001

**Fig. 4:**
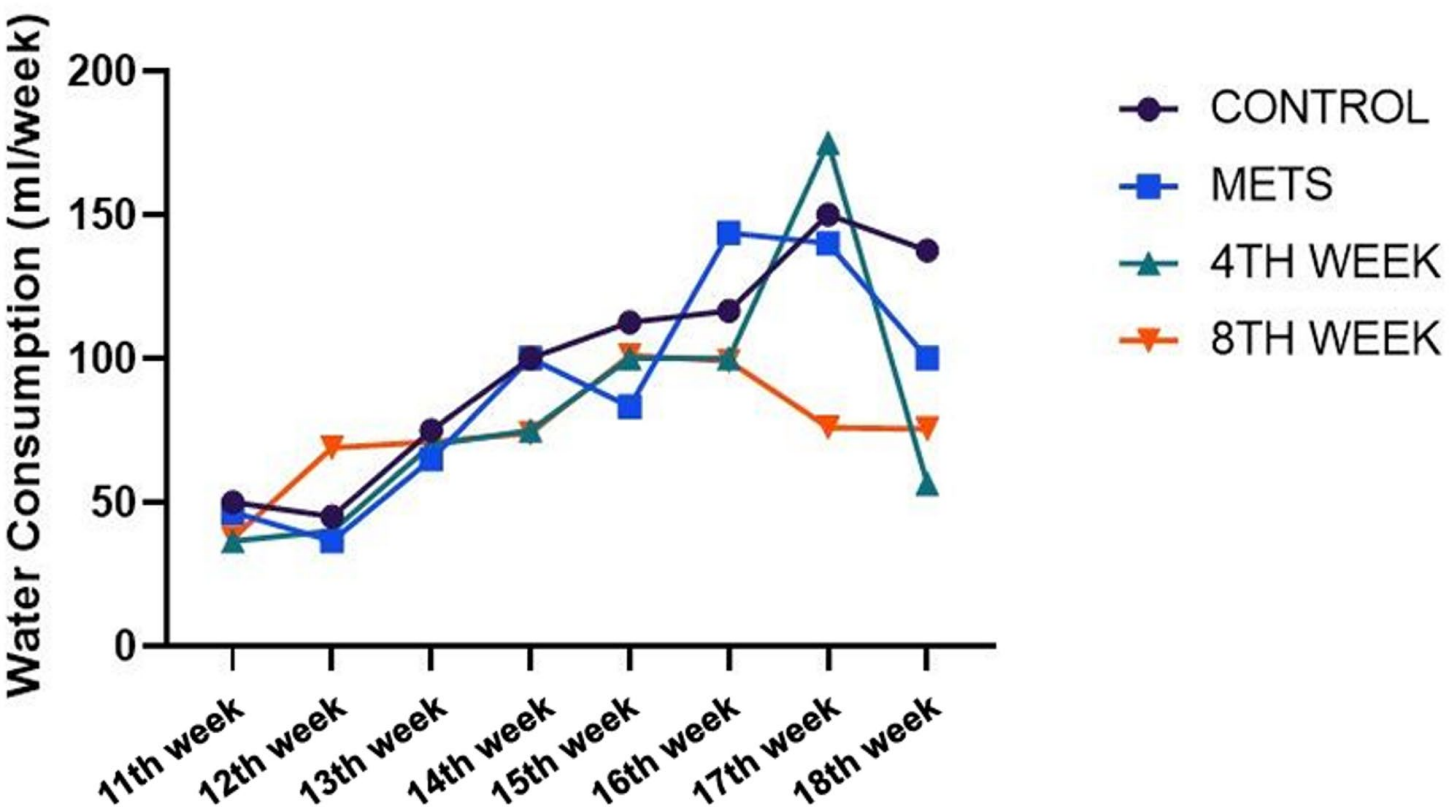
Evaluation of water consumption among groups. Data are represented as mean + SD. * < 0.05 ** < 0.01 *** < 0.001

Pellet consumption values are observed in Fig. 3. Overall, no differences in pellet consumption were observed between the groups. (Fig. 3). Regarding water consumption, a significant increase was observed in the 4TH WEEK group in weeks 5 and 6 compared to the baseline week (*p* = 0.0232, *p* = 0.001). Similarly, in the 8TH WEEK group, a significant difference in water consumption was detected between baseline and week 6 (*p* = 0.007). Although an increase in water consumption was noted within other groups over time, no statistically significant difference was observed within groups (*p* > 0.05). Furthermore, no statistically significant difference was detected between groups in terms of water consumption (*p* > 0.05) (Fig. 4).

### Evaluation of AST and ALT parameters

**Fig. 5:**
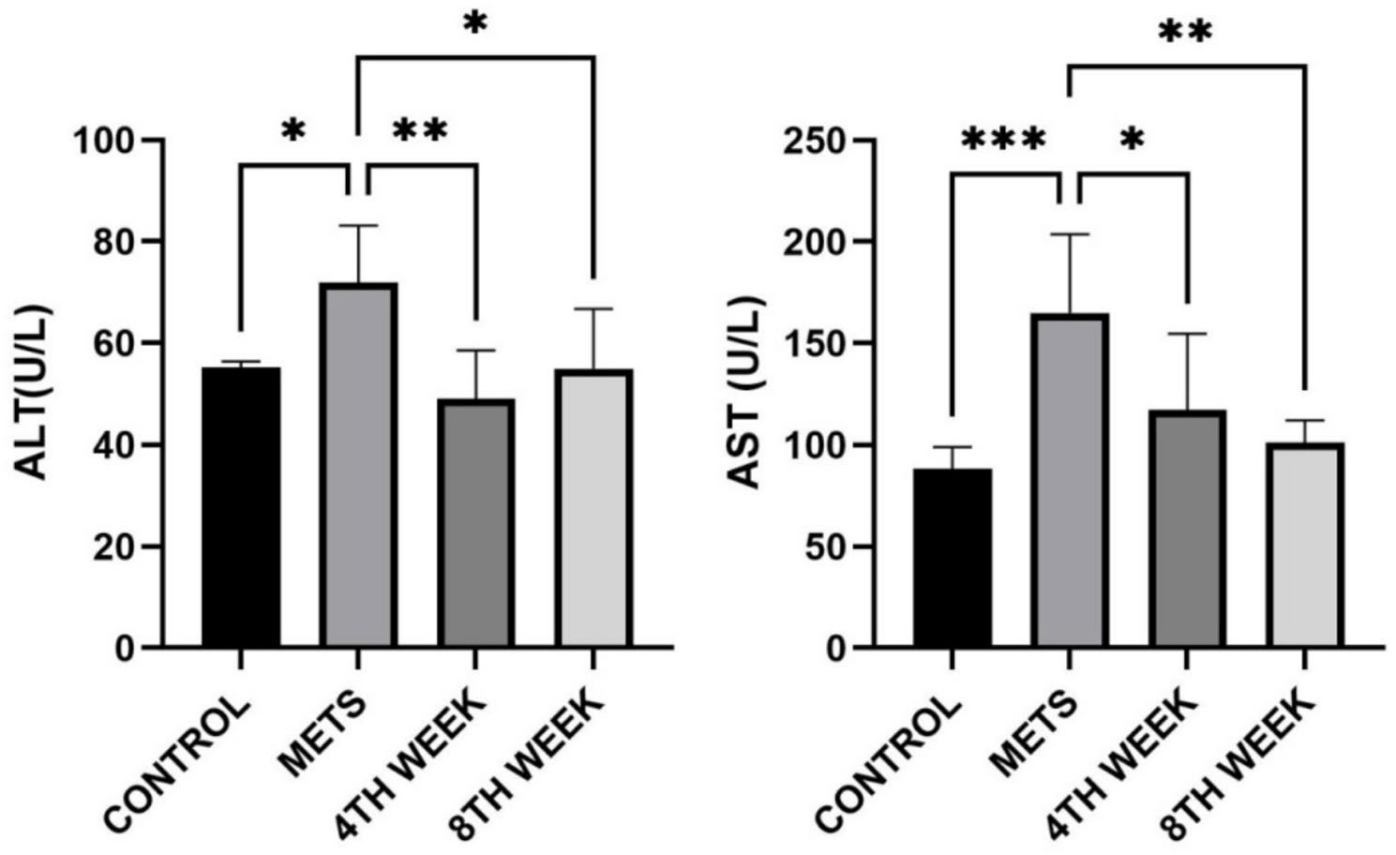
Evaluation of AST and ALT levels among groups Data are represented as mean + SD. * < 0.05, ** < 0.01, *** < 0.001

Serum aspartate aminotransferase (AST) levels in the METS group (165 ± 38.68 U/L) were significantly higher than those in the control group (88.39 ± 10.61 U/L) (*p* < 0.001). In the 4TH WEEK group, AST levels (117.2 ± 37.47 U/L) showed a significant decrease compared to the METS group (*p* = 0.036). Similarly, in the 8TH WEEK group, AST levels (101.4 ± 10.65 U/L) exhibited a marked reduction compared to the METS group (*p* = 0.004). However, no significant difference was observed between the 4TH WEEK and 8TH WEEK groups. Serum alanine aminotransferase (ALT) levels were also significantly elevated in the METS group (71.93 ± 11.18 U/L) compared to the control group (55.22 ± 1.16 U/L) (*p* = 0.028). In the 4TH WEEK group, ALT levels (49.01 ± 9.517 U/L) showed a significant decrease compared to the METS group (*p* = 0.002). Similarly, in the 8TH WEEK group, ALT levels (54.94 ± 11.76 U/L) exhibited a significant reduction compared to the METS group (*p* = 0.025). However, no significant difference was detected between the 4TH WEEK and 8TH WEEK groups regarding ALT levels. These findings indicate that hirudotherapy has a reducing effect on both AST and ALT levels in rats with metabolic syndrome (Fig. 5).

### Evaluation of lipidemic parameters

**Fig. 6:**
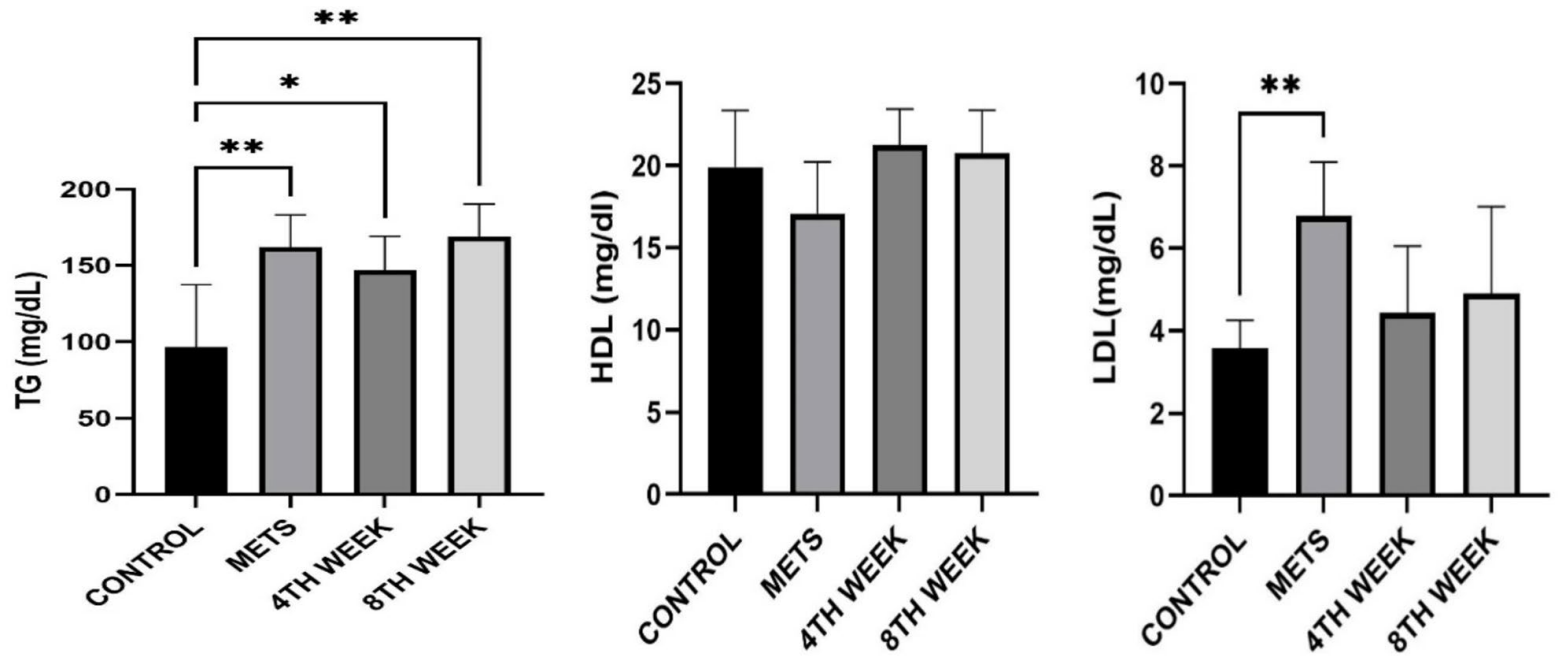
Evaluation of TG, HDL, and LDL cholesterol levels among groups. Data are represented as mean + SD. * < 0.05 ** < 0.01

Serum triglyceride (TG) levels in METS-induced rats (162.1 ± 21.22 mg/dL) were significantly higher than those in the control group (96.81 ± 40.56 mg/dL) (*p* = 0.003). In the 4TH WEEK group, TG levels (146.7 ± 22.57 mg/dL) showed a significant increase compared to the control group (*p* = 0.026). Similarly, in the 8TH WEEK group, TG levels (168.8 ± 21.61 mg/dL) were also significantly different from the control group (*p* = 0.01). However, statistical analysis within the 4TH WEEK and 8TH WEEK groups did not reveal a significant decrease in TG levels. Regarding HDL cholesterol levels, no statistically significant differences were observed between the groups (*p* > 0.05). LDL cholesterol levels in the METS group (6.78 ± 1.31 mg/dL) were significantly higher than those in the control group (3.57 ± 0.67 mg/dL) (*p* = 0.008). However, no significant difference was found between the 4TH WEEK group (4.43 ± 1.61 mg/dL) and the 8TH WEEK group (4.89 ± 2.11 mg/dL) (*p* > 0.05) (Fig. 6).

### Evaluation of hemodynamic parameters

**Fig. 7:**
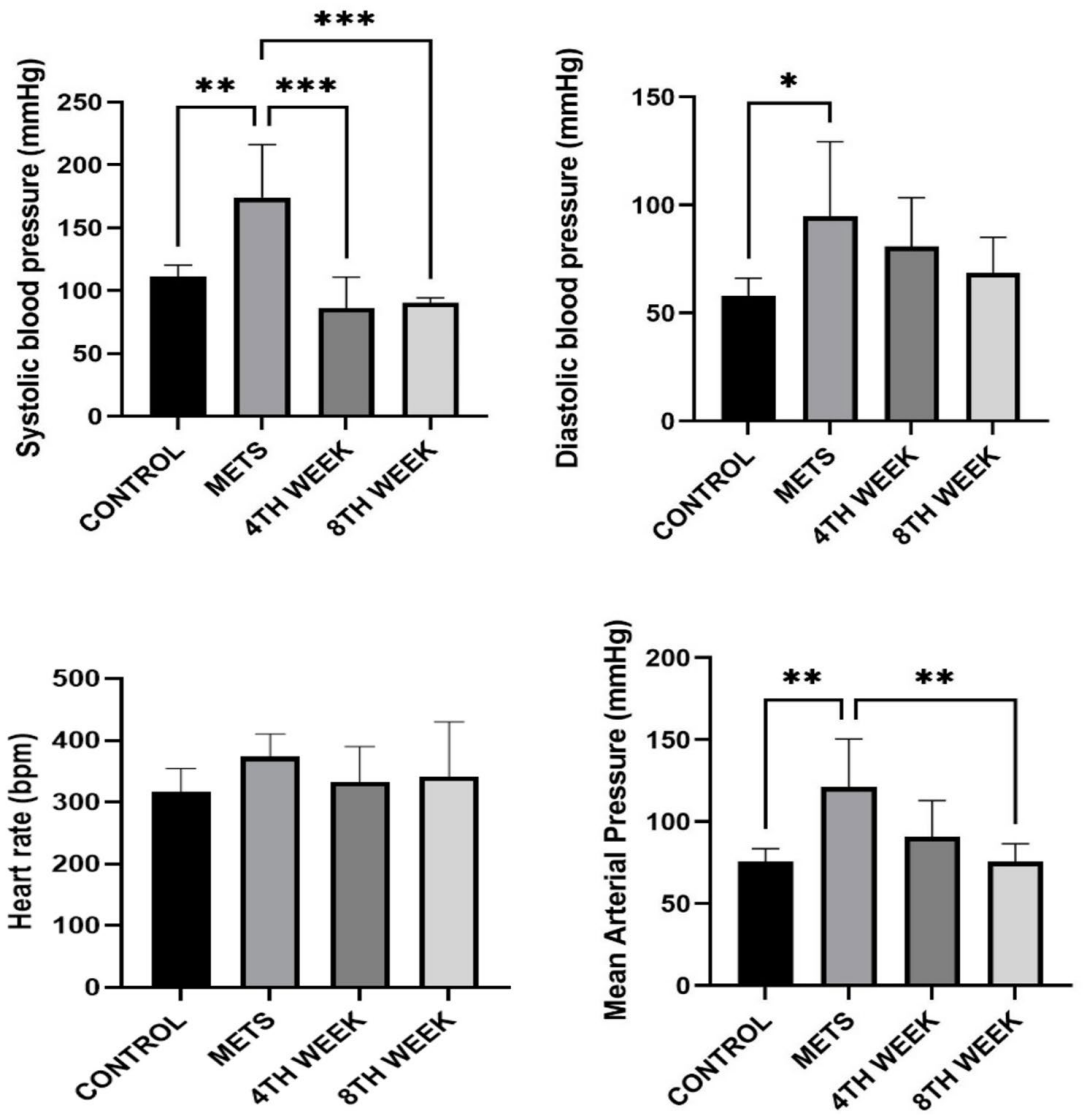
Evaluation of hemodynamic parameters among groups. Data are represented as mean + SD. * < 0.05 ** < 0.01 ***< 0.001

When examining systolic blood pressure, the METS group (174.0 ± 42.16 mmHg) exhibited significantly higher values compared to the control group (111.4 ± 8.84 mmHg) (*p* = 0.002). In the 4TH WEEK group, systolic blood pressure (86.05 ± 24.78 mmHg) showed a significant reduction compared to the METS group (*p* < 0.001). Similarly, in the 8TH WEEK group, systolic blood pressure (90.28 ± 3.93 mmHg) demonstrated a significant decrease compared to the METS group (*p* < 0.001). Hirudotherapy in both the 4TH WEEK and 8TH WEEK groups had a lowering effect on systolic blood pressure. Regarding diastolic blood pressure, only the METS group (94.82 ± 34.48 mmHg) exhibited a significant increase compared to the control group (58.10 ± 8.02 mmHg). However, hirudotherapy did not result in any significant changes in diastolic blood pressure values. When evaluating mean arterial pressure (MAP), the METS group (121.2 ± 29.19 mmHg) showed significantly higher values compared to the control group (75.85 ± 7.69 mmHg) (*p* = 0.003). In the 8TH WEEK group, MAP (75.83 ± 10.72 mmHg) was significantly reduced compared to the METS group (*p* = 0.003), indicating that 8TH WEEK effectively lowered the increased mean arterial pressure induced by metabolic syndrome. Regarding heart rate, no statistically significant differences were found among the groups. The heart rate of the control group (316.5 ± 37.74 bpm) did not show a notable difference compared to the METS group (374.1 ± 36.60 bpm). Although a decrease in heart rate was observed in the 4TH WEEK group (332.8 ± 57.46 bpm) and the 8TH WEEK group (342.0 ± 88.34 bpm) compared to the METS group, this change was not statistically significant (Fig. 7).

### Insulin resistance parameters

#### Oral glucose tolerance test and area under curve (AUC)

**Fig. 8:**
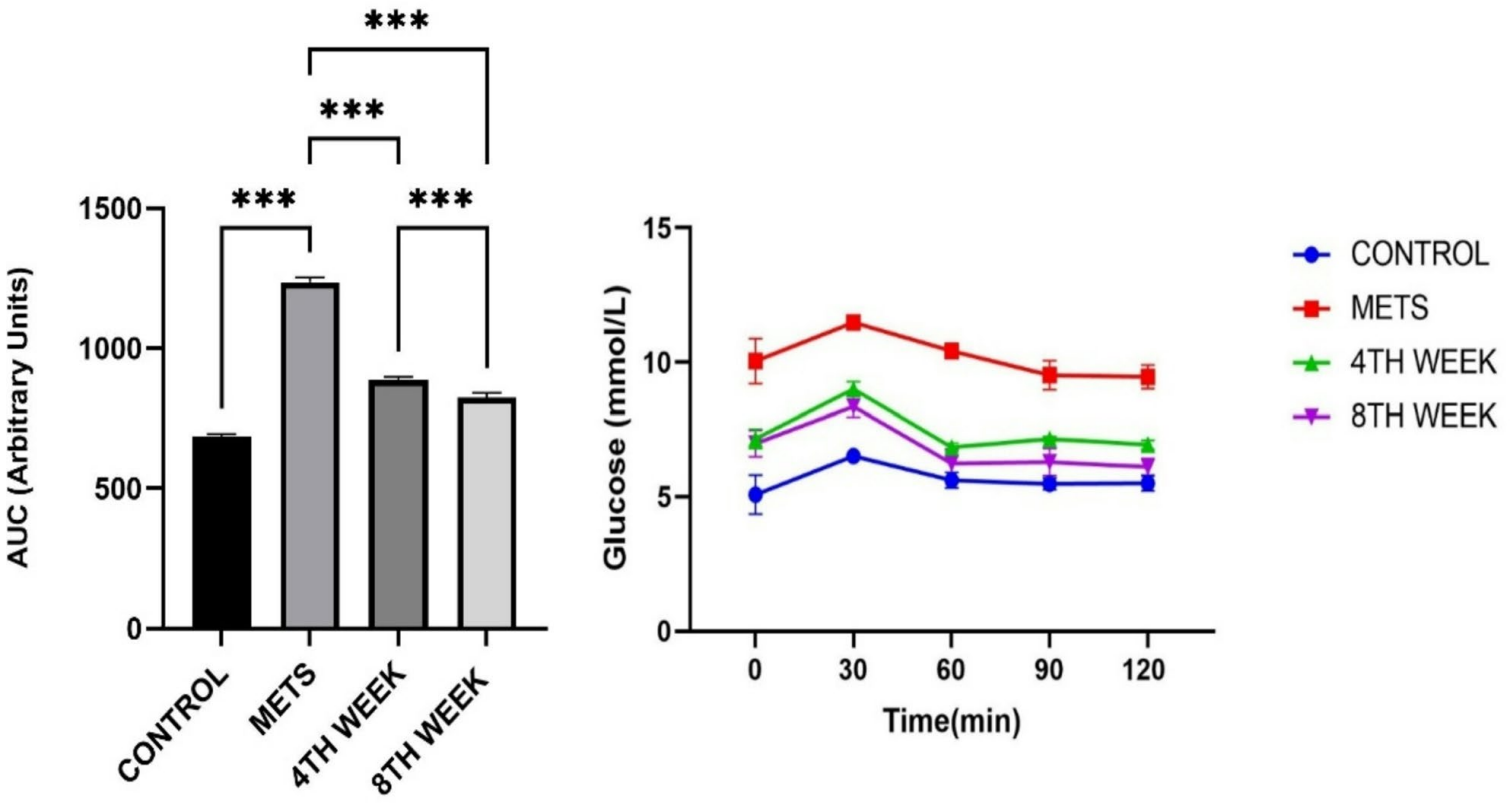
Evaluation of oral glucose tolerance test results at 0, 30, 60, 90, and 120 min and İnter-group Area Under Curve (AUC) changes. Data are represented as mean + SD. ***< 0.001

According to the Oral Glucose Tolerance Test (OGTT) results, a significant difference in baseline glucose levels was observed between the METS group and the control group (*p* = 0.002). Additionally, a significant difference was found between the 8TH WEEK group and the METS group (*p* = 0.020). At the 30-minute mark, statistically significant differences were detected between the 4TH WEEK group (*p* = 0.014) and the 8TH WEEK group (*p* = 0.016) compared to the control group. At 120 min, a significant difference was observed between the METS group and the control group (*p* = 0.003), as well as between the 4TH WEEK group and the control group (*p* = 0.003). Moreover, at 120 min, statistically significant differences were identified between the METS group and the 4TH WEEK group (*p* = 0.013), as well as between the METS group and the 8TH WEEK group (*p* = 0.006). AUC values showed significant differences among all groups (*p* < 0.001). These findings indicate that the 4TH WEEK and 8TH WEEK groups effectively reduced blood glucose levels, bringing them closer to those of the control group (Fig. 8).

### Histopathological findings

**Fig. 9:**
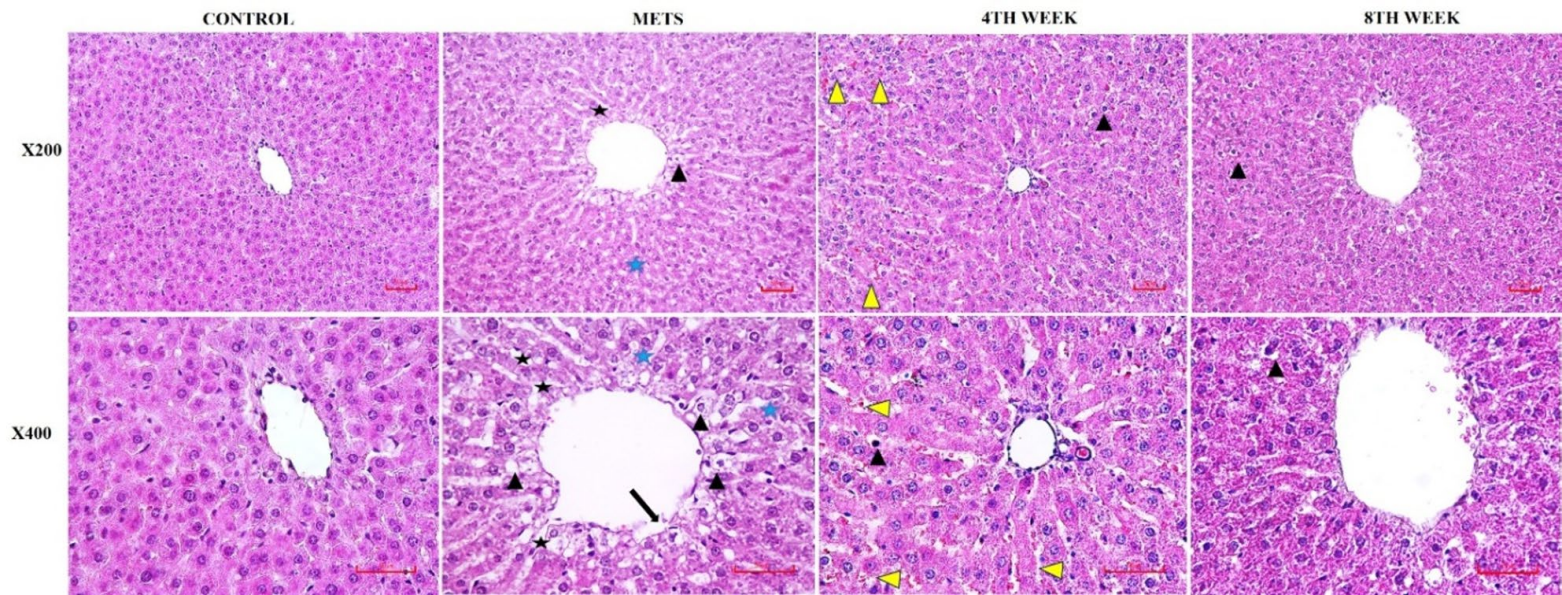
Sections of livers in diferent groups of rat. H&E (Nikon Eclipse Si, Tokyo, Japan. Scale bar; 50 μm). CONTROL group; Normal histology in control group. METS group; severe hydropic degeneration of hepatocytes (arrowheads) in the central region, sinusoidal dilatation (black asteriks) irregularities (black arrow) and vacuolization (fat vacuoles) in cords (blue asteriks), 4TH WEEK group; minimal hydropic degeneration of hepatocytes in the periacinar region. The central vein and sinusoids appear normal, with areas of hemorrhage in places (yellow arrowheads). 8TH WEEK group; liver sections in both the 8TH WEEK group and the control group appear similar

In the control group, liver tissues displayed a normal histological architecture, with hepatocytes arranged in orderly cords radiating around the central vein (vena centralis). In contrast, the METS group, in which metabolic syndrome was induced, showed prominent hydropic degeneration, increased cytoplasmic vacuolization, and dilatation of the sinusoids, particularly surrounding the central vein. Notably, extensive lipid vacuole accumulation was also observed in this group. In the 4TH WEEK group, a significant reduction in the number of hepatocytes exhibiting hydropic degeneration was evident, and the sinusoidal structures appeared more organized, closely resembling the histological appearance of the control group. The central vein regained its regular structure, and the periacinar region was surrounded by healthy hepatocytes. Furthermore, lipid vacuoles were significantly reduced in this group; however, an increase in hemorrhagic foci was noted in some sections. Both hirudotherapy groups exhibited similar histological features. In the 8TH WEEK group, sinusoids appeared more organized, the central vein exhibited normal histological characteristics, and lipid vacuoles were significantly reduced. However, in both hirudotherapy groups, the presence of hemorrhagic foci remained a noteworthy observation (Fig. 9; Table 1).

**Table 1:** Histopathological scoring table in liver tissue

	CONTROL	METS	4TH WEEKS		8TH WEEKS
Hydropic degeneration of hepatocyte	0	3	1	0	
Dilatation of sinusoids	0	3	0	0	
İrregularities in central region	0	3	0	0	
Fat vacuoles	0	3	1	1	
Hemorrhage	0	3	2	2	

none = 0, mild = 1, moderate = 2, severe = 3

## Discussion

Metabolic syndrome (METS) is a complex metabolic disorder characterized by the simultaneous occurrence of obesity, insulin resistance, dyslipidemia, and low-grade inflammation, and it is considered one of the key determinants of cardiovascular diseases [15]. Sedentary lifestyle, high-energy diet consumption, and modern living practices increase the prevalence of METS across all age groups [16]. Although current treatment strategies primarily focus on lifestyle modifications and pharmacotherapy, the inability of these approaches to produce sufficient therapeutic responses in all individuals has highlighted the importance of investigating the potential effects of complementary medical practices on METS [17]. In this context, the regulatory effects of hirudotherapy, a traditional biotherapeutic method [18], on metabolic, inflammatory, and vascular processes have drawn increasing attention in recent years.

In this study, the effects of hirudotherapy applied for 4 and 8 weeks on physiological, metabolic, and histological parameters were evaluated in a METS model induced by a high-fat and high-fructose diet. The disruption of energy homeostasis caused by the diet, leading to weight gain, hyperphagia, and metabolic stress, has been extensively described in the literature [19]. In our study, the increases in body weight and pellet consumption observed in all groups except the control group confirm the capacity of the diet to induce metabolic stress and demonstrate the successful establishment of the METS model. These findings align with the knowledge that energy imbalance, particularly associated with impaired hypothalamic appetite-regulating pathways and increased inflammatory response, plays a dominant role in HCD + HFD models. Moreover, the observed differences in pellet and water consumption in the groups receiving hirudotherapy for 4 and 8 weeks compared to the METS group indirectly support the mitigating effects of hirudotherapy on systemic inflammation and metabolic stress.

LS contains a wide repertoire of bioactive molecules secreted during feeding that exert regulatory effects on numerous host physiological pathways. Indeed, genomic and transcriptomic analyses have demonstrated that hundreds of salivary proteins are expressed in *H. medicinalis*,* H. orientalis*, and *H. verbana*, and that these proteins are not limited to classical anticoagulant or anti-inflammatory components but span multiple functional groups [20]. This repertoire includes potent anticoagulant molecules such as hirudin, calin, saratin, and apyrase; anti-inflammatory proteins such as eglins and bdellins; hyaluronidase and various metalloproteases involved in extracellular matrix remodeling; as well as diverse bioactive components including CRISP family proteins, cystatins, PAN/apple domain proteins, α2-macroglobulins, and antimicrobial peptides. This molecular diversity enables the simultaneous regulation of multiple physiological processes, such as the transient modulation of hemostasis, suppression of inflammatory signaling, enhancement of microcirculation, and preservation of tissue integrity [21].

Indeed, the literature reports that LS suppresses the production of pro-inflammatory cytokines such as TNF-α, IL-6, GM-CSF, and IL-12p40, and reduces macrophage-mediated inflammatory responses [22]. This mechanism is consistent with the observed improvements in liver injury markers. The marked recovery observed in histopathological findings following both 4-week and 8-week treatments indicates a healing process in which the inflammation–oxidative stress cycle is disrupted and hepatic regeneration is supported. The anticoagulant, protease inhibitory, and microcirculation-regulating properties of molecules found in LS—such as hirudin, bdellin, eglin, and destabilase—provide a scientific basis for explaining this pattern of hepatic improvement [23–25]. Additionally, recent fibroblast models have shown that LS promotes tissue regeneration by enhancing growth factor expression and cell migration [26], which is consistent with the regenerative response observed in the liver.

The improvement in lipid metabolism is consistent with LS’s capacity to reduce inflammatory burden and enhance circulation. The decrease in TG and LDL cholesterol levels can be explained by the alleviation of oxidative stress and inflammation, which play key roles in the pathogenesis of dyslipidemia. In MSG (Monosodium Glutamate) or diet-induced dyslipidemia models, increased lipid peroxidation and elevated inflammatory cytokines have been reported to promote lipid accumulation [27, 28]. LS’s ability to suppress inflammatory signals, regulate endothelial function, and enhance microcirculation aligns with the normalization of the lipid profile observed in our study. In this context, apyrase, hyaluronidase, and various metalloproteases present in LS have been shown to modulate the extracellular matrix, thereby increasing tissue perfusion and consequently reducing oxidative stress burden; transcriptomic analyses further indicate that CRISP and cystatin family proteins may contribute to the mitigation of inflammation-induced metabolic load through their immunomodulatory properties [20]. These mechanisms support the molecular basis of the reductions in TG and LDL cholesterol observed in our study. Additionally, LS has been reported to exert regulatory effects on metabolic pathways such as PI3K/Akt, PKC, NF-κB, and PLCG2, and these molecular actions may contribute to the attenuation of dyslipidemia and hepatic steatosis [24, 25].

The improvement observed in hemodynamic parameters suggests an attenuation of endothelial dysfunction, which is one of the fundamental components of METS pathophysiology. In METS models, vascular inflammation and reduced nitric oxide (NO) bioavailability lead to elevated blood pressure [29]. The ability of hirudin and similar anticoagulant molecules in LS to enhance microcirculation, reduce inflammation, and alleviate endothelial stress is consistent with the decrease in blood pressure observed in our study. In addition, anticoagulant proteins such as saratin, calin, and antistasin found in LS have been reported to inhibit platelet adhesion and the coagulation cascade, while M12-type metalloproteases modulate extracellular matrix components and contribute to maintaining blood fluidity within tissues [21]. When evaluated together with these biochemical effects—facilitating microcirculation and reducing vascular load—the improvements in hemodynamic parameters observed in our study are supported by a strong mechanistic basis.

The favorable effects observed on glucose metabolism are associated with the potential of LS to enhance insulin sensitivity. Improvements in OGTT and AUC analyses are consistent with the positive contributions of the antioxidant and anti-inflammatory capacity of LS to glucose homeostasis [30]. Moreover, gliarin, NGDF-like proteins, and various potassium channel subunits identified in LS have been reported to modulate signaling pathways such as NF-κB, PI3K, JNK, PKC, and PLC [22]. In addition, molecules such as α2-macroglobulin, cystatins, CRISP proteins, and adenosine deaminase identified in the reported salivary proteome have been shown to play roles in regulating immune responses and inflammation, and these regulatory effects are proposed to indirectly contribute to the alleviation of inflammation-induced metabolic stress; hyaluronidase and metalloproteases, by modulating the extracellular matrix, have been reported to increase tissue perfusion, thereby creating a microenvironment that may facilitate glucose utilization [20]. These mechanisms are consistent with the improvements in glucose tolerance observed in our study.

Histopathologically, the marked improvement observed in hydropic degeneration, sinusoidal dilatation, and lipid vacuoles within hepatocytes demonstrates the supportive effect of LS on tissue regeneration. Despite persistent hemorrhagic foci, the restoration of tissue integrity following the 8-week treatment is consistent with literature reports indicating that suppression of oxidative stress can accelerate the regeneration process [31]. The anticoagulant (hirudin, calin), anti-inflammatory (eglin, bdellin), and fibrinolytic/destabilase components of LS are known to enhance microcirculation and improve perfusion in hypoxic tissues, contributing to the restoration of sinusoidal integrity and the normalization of energy metabolism in hepatocytes. Furthermore, hyaluronidase and various metalloproteases facilitate tissue remodeling by modulating the extracellular matrix, while CRISP and cystatin family proteins have been shown through transcriptomic and proteomic studies to limit inflammatory cell infiltration and support the regenerative process within the hepatic microenvironment [20]. This multifaceted biological activity profile suggests that the histopathological improvement observed in our study may be attributable not only to the reduction of inflammation but also to LS components that directly modulate tissue repair and cellular renewal processes.

## Conclusion

In conclusion, hirudotherapy appears to exert multifaceted regulatory effects on inflammation, oxidative stress, lipid metabolism, glucose homeostasis, and vascular function, all of which constitute fundamental components of METS pathophysiology. This multidimensional effect profile is consistent with the anticoagulant, anti-inflammatory, microcirculation-enhancing, and metabolic pathway–modulating properties derived from the rich repertoire of bioactive components in medicinal leech saliva. However, the limitation of the study to only 4- and 8-week treatment periods restricts the evaluation of long-term metabolic and histological effects. Moreover, the specific mechanisms of action of the molecular components of LS have not yet been fully elucidated, indicating that certain uncertainties persist regarding clinical translation. Therefore, mechanistic studies investigating both longer-term treatment protocols and the effects of specific molecules within LS on target pathways are required. Based on the current findings, although hirudotherapy holds potential as a complementary approach for metabolic and inflammatory disturbances associated with METS, more comprehensive data on safety and efficacy are needed before clinical application can be fully established.

## Data Availability

All data included in the manuscript are available.

## Abbreviations

METS: Metabolic syndrome
AST: Aspartate aminotransferase
ALT: Alanine aminotransferase
TG: Triglyceride
HDL-C: High-density lipoprotein cholesterol
LDL-C: Low-density lipoprotein cholesterol
OGTT: Oral glucose tolerance test
AUC: Area under the curve
MAP: Mean arterial pressure
HFD: High-fat diet
HCD: High-carbohydrate diet
LS: Leech saliva
